# Learning by doing in practice: a roundtable discussion about stakeholder engagement in implementation research

**DOI:** 10.1186/s12961-017-0275-8

**Published:** 2017-12-28

**Authors:** Said Habib Arwal, Bhupinder Kaur Aulakh, Ahmed Bumba, Akshita Siddula

**Affiliations:** 1 Community Based Health Care Department, Ministry of Public Health, Kabul, Afghanistan; 2 Health and Family Welfare, State Government of Uttarakhand, Dehradun, Uttarkhand India; 3 Kibuku District Government, Kibuku, Uganda; 4 Department of International Health, Johns Hopkins University, Baltimore, MD United States of America

**Keywords:** Afghanistan, India, Uganda, Stakeholder engagement

## Abstract

**Background:**

Researchers and policy-makers alike increasingly recognise the importance of engaging diverse perspectives in implementation research. This roundtable discussion presents the experiences and perspectives of three decision-makers regarding the benefits and challenges of their engagement in implementation research.

**Discussion:**

The first perspective comes from a rural district medical officer from Uganda and touches on the success of using data as evidence in a low-resource setting. The second perspective is from an Afghani Ministry of Health expert who used a community-based approach to improving healthcare services in remote regions. Finally, the third perspective highlights the successes and trials of a policy-maker from India who offers advice on how to grow the relationship between decision-makers and researchers.

**Summary:**

Overall, the stakeholders in this roundtable discussion saw important benefits to their engagement in research. In order to facilitate greater engagement in the future, they advise on closer dialogue between researchers and policy-makers and supporting the development of capacity to stimulate and facilitate engagement in research and the use of evidence in decision-making.

## Background

Understanding and engaging stakeholders in health systems research has been recognised as important for aligning research and policy agendas, increasing buy-in for the implementation and scale-up of evidence-based interventions, as well as for facilitating evidence-informed decision-making [1–3]. Researchers can find important stakeholders to engage across all levels of a health system, from policy-makers and healthcare professionals, to community members. Much has been written about community-based participatory research and action research [4], and there is also a burgeoning literature on knowledge translation and how best to engage policy and decision-makers in research processes [5]. However, it is much less common for the perspectives of such stakeholders, including policy-makers, mid-level managers and health workers, to be directly represented in the literature.

In this roundtable discussion, we highlight what engagement of stakeholders in research looks like from the perspectives of three stakeholders that have been a part of implementation research projects and how they have used the resulting evidence in their day-to-day work to make decisions. This roundtable discussion highlights the various roles each stakeholder plays within their respective health systems and how they have been able to use implementation science to change the design and operation of their programmes and to strengthen internal processes.

We start with a district medical officer from Uganda, Dr Bumba, who was closely involved in implementation research conducted by Future Health Systems that sought to improve the quality and accessibility of maternal and neonatal health services. We then move onto a contribution from an Afghani public health professional, Dr Arwal, who recounts the role of implementation research in his success in implementing a community based approach to healthcare in rural and conflict ridden communities. In particular, he reflects on how a Future Health Systems project on community scorecards helped connect local stakeholders, and the role that an implementation research approach played in fine tuning the scorecard intervention. Finally, a policy-maker from India, Dr Aulakh, discusses the many obstacles facing evidence-based decision-making in a developing country and offers guidance on how to overcome these challenges.

## Using data as evidence in a rural context

### Dr Ahmed Bumba – District Health Officer, Kibuku District Government, Kibuku, Uganda

Rural settings provide distinct challenges in implementing research that uses evidence and data for decision-making. As a district medical officer, my day-to-day work provides unique opportunities to learn by doing and adapt research to a rural context. In the Kibuku district of Uganda there is a heavy burden of illness from communicable disease as well as a growing prevalence of non-communicable diseases due to increasing access to alcohol, cigarettes and processed foods. Our health system is designed to be curative in nature with just a small aspect focused on prevention. The greatest portion of the budget is allocated to pharmaceuticals, with less than 1% of the budget geared towards prevention. As a result, the health facilities are burdened with a huge number of cases.

Working in our communities, it has been clear that there are gaps in service delivery. The Future Health Systems partnership helped enhance our management capabilities to improve the health system. We learned that we already have some structures, resources and opportunities within the district that can be used to address the service delivery gaps. For example, we have always had transporters (boda boda drivers) in the district and community health workers (CHWs), but we had not thought about connecting them before, and by making this connection we have been able to help pregnant women get to health facilities more easily. At the forefront of this change in health systems improvement is trial and error. We have learnt to start small, see if a positive impact can be made, and then learn from the intervention to improve at a larger scale.

We have started doing things that have not been done before, such as using data for decision-making. Our districts have a wealth of data that is collected on a daily basis, which we submit to the Ministry, but is never used at the local level. Working with Makerere University helped us figure out how to use the data as evidence for decision-making at the health facilities.

We now conduct quarterly meetings to review the collected data and use the numbers to help us decide how to allocate resources. This data-driven process can be applied to many decisions regarding interventions, human resource allocation, immunisation intervention and outpatient organisation. For example, the data showed that weekly market days in the community result in a sharp increase in outpatient attendance, which led us to reorganise staff to ensure we are adequately staffed on market days. Reviewing such data has helped us use the few resources we have in a more directed manner to address the gaps we see. As we grow our capacity as a district to use data, we see the importance of interrelationships between the different players involved in the delivery of health. This has led to the re-energising of the district structure, which helps us work together and contribute to each other’s efforts.

A key challenge we have faced in implementing changes to public service delivery is complacency. The general attitude is to do business as usual, which has led to a lack of innovation. Those who have tried to work outside of the box are negatively perceived for challenging the status quo. We are trying to change the way health staff think about innovation. Another problem is that we have quite a limited capacity to do our own data analysis. We have just one biostatistician in the district, and they would really benefit from having more skills.

Another challenge that we face concerns a lack of resources. I do not like to say that limited resources are a challenge but, for example, the shortage of vehicles in the district is a problem for effective supervision. As a manager, it is imperative that I allocate the resources that we have appropriately and make decisions based on the data for the benefit of the system as a whole. For example, we are now working to address maternal and child health by building a system where we focus on the most vulnerable. We are identifying high-risk mothers, young mothers and mothers who have never delivered in a health facility with previous pregnancies, and we are tracking them with CHWs who help connect them to the health facilities closest to them.

Working with Makerere University, I have realised that working within one’s comfort zone is not enough to lead to sustainable change in the system. Working with the data has broadened our expectations of ourselves and of the health system, driving us to make innovative new improvements and build on the capacity of our team to deliver a quality service.

## Engaging communities in strengthening health systems

### Said Habib Arwal – Director of Community Based Health Care Department, Ministry of Public Health, Afghanistan

As a doctor, you only ever get to practice medicine by treating one person at a time. Public health, on the other hand, is about taking care of a community. It is this community-based approach that drew me to my work when I first started at the Ministry of Public Health. Under former President Karzai, Afghanistan started to rehabilitate its health system and address the shortage of health staff in rural provinces. We soon established the Community Based Health Care Department in 2005, with the goal of having the government work in partnership with the communities it serves as well as with non-governmental organisations (NGOs) that are providing healthcare services. The Ministry of Public Health strived to engage with community members and researchers to identify and invest in interventions that were consistent with government priorities.

As a result, the community scorecard project was launched, with support from the Future Health Systems Consortium, in two provinces in rural Afghanistan. The scorecard provided community members with a mechanism for involvement and engagement with the quality improvement process of healthcare. The project served as a link between local authorities, both governmental and non-governmental health providers, and the community members receiving the services. The results were shared with a technical advisory group, which encouraged us to expand the pilot programme to another province. The next round of results was then presented to the Ministry of Public Health for approval. With the support of the Ministry, we are hoping to continue to expand implementation to additional provinces and scale up nationwide.

The objective of the scorecard was to bring about a change in community behaviour and solicit their perspective on how to improve the current health system. As the government took steps to move away from a reliance on international donor support, we turned to the community for guidance regarding priorities for improvement. We did this by getting the patients themselves interested in the health system. The scorecards gave them the opportunity to think critically and reflect on how to make changes. Some communities wanted waiting areas at the clinics while other communities wanted latrines made available at their local health facilities.

The act of using the scorecard empowered the community not only to suggest changes but also ensured accountability from the health system so that the necessary steps were taken to implement these changes. In one community, patients expressed concerns about the lack of female staff. Both the health facility staff and the community members worked to identify the barriers preventing female staff from joining the clinic, such as the lack of housing for female staff. They worked together to come up with a multi-pronged solution with the community, opening a home for female health workers, the NGO agreeing to provide for the salary of a new staff member and the local government agreeing to provide security to ensure the safety of the new female staff.

This three-way partnership is essential for the sustainability of this scorecard programme and the health system at large. The implementation of community scorecards, with the support of Future Health Systems, helped to facilitate this partnership. With the government, local NGOs, and communities all coordinating together, we now have a stronger investment in the system. This cooperation is a key element of health services in Afghanistan, a country where close to 30,000 CHWs work closely with community stakeholders to address health promotion and education. In times of conflict and instability, it is difficult to convince people of the benefit of a health programme or of seeking care at a clinic. With our CHWs, we strive to work with the community, by the community and for the community.

There are many challenges when it comes to this work. Political insecurity and rampant poverty are major issues in Afghanistan. CHWs are all young volunteers, many of them women, who are working in insecure areas with support from both the government and NGOs. Even in an insecure area with no government, our volunteer health workers are able to provide a service because they are from that community. Despite Taliban rule, CHWs can keep health facilities open and functioning.

Working with the Future Health Systems project, we have been able to implement the community scorecards – a monitoring tool that can be used to engage the public and service providers in quality assurance and accountability. The opportunity for providers to interface with the community for feedback serves to empower both patients and physicians. The technical quality of services is better assessed, utilisation rates have increased and we are better able to understand the barriers that exist with regards to seeking care. The community is telling us directly what they need and what works for them, thus working with the data collected from the community scorecards has helped our health system grow and adapt to the needs of the community and better serve our people.

## Making evidence-based decisions as a policy-maker

### Dr Bhupinder Kaur Aulakh – Secretary, Health and Family Welfare, State Government of Uttarakhand, India

As Secretary of Health and Family Welfare for the state government of Uttarakhand in India, I served as a senior policy-maker working under the Minister of Health. In this role, I was responsible for developing policies and presenting them for approval, and once approved, I worked to ensure they were properly implemented. As a policy-maker, I rely on evidence to direct our policy development and to identify solutions for possible interventions.

Evidence-based decision-making is very important to us. However, there are many differences between the world of research and the real world. A mutual understanding of health research among researchers and policy-makers does not always exist. Researchers want to share their results but they should be presented in a way that can be easily implemented. People in academic institutions and in universities have little understanding of how policy impacts practice. Policy-makers are more than willing to use research in their area of work.

In a low-income country like India, we do not have a wealth of health research as it is traditionally understood. In government, we have routine data that is collected on a regular basis. The research generated by these smaller programmes within the state can lead to equally effective change on a policy level. The availability of this research differs between states, as some higher-performing states have strong surveillance systems and therefore more information to work with. As a policy-maker, I have also collected data myself, looking at distributions of health services and health outcomes.

For example, in 2016, we worked on a malnutrition intervention to address the social determinants of health. We conducted a survey of the entire state to determine levels of severe and moderate acute malnutrition among young children and found significant numbers of malnourished children. Our first step in addressing this problem was to consider the existing research on how to treat malnutrition. I then worked to adapt the research findings to what was feasible within our context and within our available budget. The state allocated six rupees per child for food supplements and, within that limit, we devised a therapeutic food based on locally available and locally grown food grains. We relied on additional research on how to best use these grains and worked with a local agricultural school to determine the shelf life of the final supplement product. All this research led to the creation of a millet-based food supplement that was distributed to families of malnourished children. We then started following a group of 20–25 children over time, measuring their growth every month from September to March. We documented their weight gain and saw them rise out of malnutrition status. We then shared the results of this small programme with the Indian government and the recommendations were then shared with all other states to shape best practices in addressing acute malnutrition.

There are many challenges that exist in implementing research findings, especially in low-income settings. There are few university counterparts in smaller states to work with regarding implementation research, leaving policy-makers working alone. Existing systems often do not have the capacity to immediately implement the solutions as they exist. Research findings need to be adapted and modified to be context specific. Often, there is a time constraint and a lack of trained personnel or funding needed to follow through on these findings. These are the issues that policy-makers grapple with as we work to translate research into policy.

Additionally, the research capacity to implement policy-relevant research is not always there, and when it is, researchers do not always ask policy-makers to be engaged in research in the formative stage or in the implementation, but more of such partnerships would be mutually beneficial. There is a lot to be done to address this gap. Policy-makers are at the forefront of implementation. They should have access to the latest research and evidence-based best practices. National, Ministry of Health-led conferences, where researchers and policy-makers come together and research results are shared, are a helpful way to begin the dialogue. Furthermore, while there are channels for sharing local research through annual conferences, policy-makers lack access to cross-country research. Though some of this research is available through online platforms, policy-makers often lack the time needed to comb through published research. They need ready-to-use and easily digestible research findings to solve issues on hand, which is often not available.

With regards to implementation of research findings, we, as policy-makers, are looking for direct communication and information that is easy to digest. It is important that researchers work to distil research findings into easy recommendations and present results so that policy-makers can easily consume the information to make evidence-based decisions. Universities and large research groups should be reaching out to policy-makers at state and national levels to engage them in discussions and to determine priorities to address through research. Participatory engagement of policy-makers in the research process is important for facilitating the use of the evidence generated into policy formulation and implementation.

## Summary

All three of our roundtable participants recognised the benefits of their engagement in research. In particular, it is notable how, in the eyes of the policy-makers and practitioners, implementation research provided a means to break down siloes between different groups and convene multiple actors such as community members, healthcare providers and researchers. However, in practice, challenges to engagement persist, including gaps in capacity on both the policy-maker and research sides, a shortage of both time and financial resources to participate in research activities and to implement at scale what is recommended by research, and with regards to system-wide barriers in terms of contextual challenges and lack of incentives for innovation and large-scale change. Direct lines of communication between researchers and decision-makers are important for turning research findings into policy recommendations that are easy to understand and incorporate into implementation plans. More frequent partnerships between researchers and policy stakeholders would be facilitated by increasing the opportunity for dialogue between these two groups, supporting the development of capacity to facilitate engagement in research and the use of evidence in decision-making.
